# Supplementary material for: Extra-Thyroidal Impacts of Serum Iodine Concentrations During Early Pregnancy on Metabolic Profiles and Pregnancy Outcomes: Prospective Study Based on Huizhou Mother–Infant Cohort
Source: Nutrients. 2025 May 9;17(10):1626. doi: 10.3390/nu17101626 (PMC12113788; doi:10.3390/nu17101626)
Supplement: Supplementary file 1 [file nutrients-17-01626-s001.zip › nutrients-3585834-supplementary.pdf]

Supplemental Table S1 Stratification analyses by SIC less than and above 88.9 ug/L according to the results of non-linearity tests by RCS, Huizhou Mother-infant Cohort

		Crude model				Model 1				Model 2 (+ thyroid markers)		
	n	*B*(95% *CI*)	*β*	*P*		*B*(95% *CI*)	*β*	*P*		*B*(95% *CI*)	*β*	*P*
**1-hr PG, mmol/L**												
**SIC<88.9μg/L**	745	0.772(-2.210, 3.754)	0.024	0.611		1.576(-1.293, 4.445)	0.049	0.281		1.947(-0.927,4.821)	0.061	0.184
**SIC⩾88.9μg/L**	711	1.256(-2.127,4.638)	0.035	0.466		1.424(-1.830,4.679)	0.040	0.390		2.046(-1.444,5.535)	0.057	0.250
**2-hr PG, mmol/L**												
**SIC<88.9μg/L**	745	0.458(-2.061, 2.977)	0.017	0.7213		1.294(-1.094, 3.683)	0.048	0.287		1.628(-0.768, 4.023)	0.060	0.182
**SIC⩾88.9μg/L**	711	1.502(-1.442, 4.446)	0.048	0.316		1.719(-1.135, 4.572)	0.055	0.237		2.096(-0.961, 5.154)	0.067	0.178
**UA, μmol/L**												
**SIC<88.9μg/L**	745	0.976(-96.12,98.07)	0.001	0.984		-8.484 (-101.3,84.38)	-0.008	0.858		-11.815(-105.51,81.88)	-0.011	0.804
**SIC⩾88.9μg/L**	711	31.788(-89.60,153.17)	0.024	0.607		29.253(-88.92, 147.43)	0.022	0.627		8.859(-117.90,135.62)	0.007	0.891

Mothers of diagnosed pre-pregnancy diabetes, hepatic or thyroid disorders or current hepatic diseases were excluded and 1456 pregnant women remained for the stratification analysis by SIC at T1 less or higher than the median of 88.9μg/L. Serum iodine concentrations (SIC) were Log_{10} transformed due to skewed distribution. Data were analyzed by multivariable linear regression with covariates being adjusted by enter method. The adjusted covariates in model 1 included, maternal age(y), pre-pregnancy BMI(kg/m^2), education (primary school and below, middle school, college, university and above), parity (0, ≥1), active or passive smoking (yes/no), alcohol drinking (yes/no), medical history of GDM (yes/no), pre-pregnancy PCOS, first-degree family history of diabetes(yes/no), total sitting time(h/d), hormones usage during pregnancy (yes/no) and gestational weeks for biochemical testing; model 2: further adjustment was made for thyroid hormones including LgTSH and LgFT3/FT4. For 2hPG and TC, pre-pregnancy BMI was additionally adjusted in model 1 and model 2. Abbreviations: PG, post-load glucose, UA, uric acid.

Supplemental Table S2 Estimated means and standard errors of serum iodine concentrations (SIC) in early pregnancy(T1) by different levels of metabolic factors using General Linear Models (GLM), Huizhou Mother-infant Cohort

	n	**Crude model**		**Model 1**		**Model 2**	
		**SIC at T1**	*p*	**SIC at T1**	*p*	**SIC at T1**	*p*
GMS number			0.711		0.405		0.013
0	782	90.286±17.412		89.435±0.660		88.470±0.601	
1-2	544	89.571±15.676		90.576±0.782		90.871±0.712*	
3-4	54	90.926±18.536		92.557±2.480		94.560±2.253*	
BMI at T1, kg/m^2			0.092		0.042		0.501
<23.0	1076	90.470±0.515		90.704±0.618		89.420±0.557	
≥23.0	336	88.797±0.848		88.136±1.085*		90.218±1.006	
Mean difference				-2.568±1.263*		0.798±1.186	
GWG at T1, kg			<0.001		<0.001		0.230
<median (0.8 kg)	727	91.632±0.623		92.144±0.751		90.286±0.681	
≥median (0.8 kg)	705	88.453±0.633		88.054±0.770		89.097±0.683	
Mean difference				-4.090±1.079		-1.189±0.990	
Body fatness %			0.761		0.071		0.007
<median (29.0%)	355	89.515±0.854		87.621±1.102		86.747±1.019	
≥median (29.0%)	354	89.883±0.855		90.758±1.088		91.065±0.998*	
Mean difference				3.173±1.733		4.318±1.599*	
GDM			0.673		0.485		0.322
No	1195	89.914±0.489		89.904±0.588		89.438±0.520	
Yes	245	90.414±1.079		90.959±1.373		90.784±1.238	
Mean difference				1.055±1.511		1.517±1.525	

	n	**Crude model**		**Model 1**		**Model 2**	
		**SIC at T1**	*p*	**SIC at T1**	*p*	**SIC at T1**	*p*
Maternal hyperlipidemia			0.220		0.497		0.065
no	265	89.776±0.495		89.905±0.593		89.210±0.526	
yes	1152	91.181±1.032		90.989±1.453		91.805±1.276	
Mean difference				1.084±1.595		2.594±1.405	
GH			0.132		0.183		0.349
no	933	90.114±0.445		90.161±0.537		89.680±0.477	
yes	22	85.353±3.128		85.309±3.597		86.667±3.176	
Mean difference				-4.852±3.640		-3.012±3.215	
TyG-index			0.040		0.035		0.002
<8.73	1092	89.784±0.509		89.821±0.603		89.273±0.539	
≥8.73	171	92.628±1.287		93.946±1.819*		94.699±1.621*	
Mean difference				4.125±1.948*		5.426±1.741*	
TG, mmol/L			<0.001		<0.001		<0.001
<median (1.15)	500	88.104±0.630		88.007±0.756		87.231±0.667	
≥median (1.15)	448	91.939±0.625		92.378±0.801*		92.278±0.706*	
Mean difference				4.371±1.129*		5.046±0.998*	
TC, mmol/L						1.615±1.870	
<median (4.16)	502	89.359±0.633	0.130	89.761±0.745	0.545	88.937±0.662	0.137
≥median	446	90.712±0.629		90.423±0.791		90.378±0.699	
Mean difference				0.662±1.092		1.441±0.969	
UA, μmol/L			0.047		0.011		0.341
<357	914	89.892±0.455		89.798±0.549		89.545±0.488	

	n	Crude model		Model 1		Model 2	
		**SIC at T1**	*p*	**SIC at T1**	*p*	**SIC at T1**	*p*
⩾357	49	93.998±2.014		96.065±2.402		91.647±2.148	
Mean difference				6.267±2.468*		2.102±2.207	
FSI			0.656		0.110		0.002
<median (12.6)	495	89.836±0.640		89.018±0.855		87.759±0.759	
≥median (12.6)	442	90.240±0.640		91.253±0.917		91.677±0.811*	
Mean difference				2.235±1.397		3.918±1.243*	

Mothers of diagnosed pre-pregnancy hepatic or thyroid disorders or current hepatic diseases were excluded and 1456 pregnant women remained for analysis. Data were analyzed by multivariable general linear model (GLM) with the mean difference being compared by LSD approach. The adjusted covariates in model 1 included, maternal age(yrs), pre-pregnancy BMI (kg/m^2), education (primary school and below, middle school, college, university and above), parity (0, 1,2, ≥3), smoking (yes/no), alcohol drinking (yes/no), medical history of GDM (yes/no), pre-pregnancy PCOS(yes/no) or thalassemia (yes/no), family history of diabetes in the first-degree relatives (yes/no), total sitting time (hrs), folate supplementation (yes/no) and hormone usages in pre- and early pregnancy(yes/no). In model 2, further adjustment was made for thyroid hormones including LgTSH and LgFT3/FT4. For BMI at T1, GWG at T1, and TyG-BMI, maternal pre-pregnancy BMI was not adjusted due to potential collinearity. For GH, additional adjustment was made for medical and family history of GH (yes/no) instead of medical and family history of GDM, respectively. Gestational metabolic syndromes (GMS) was determined with at least 3 factors among the following conditions: early pregnancy BMI ⩾23.0 kg/m^2, current gestational diabetes (GDM), current gestational hypertension (GH) or TG⩾1.7mmol/L. Maternal hyperlipidemia was defined as TC⩾5.18 mmol/L or TG⩾1.7 mmol/L. TyG-index=In[TG (mg/dL)×FBG (mg/dL) /2] with values above 8.73 suggesting insulin resistance; TyG-BMI=TyG-index ×BMI at T1. TyG-BMI was non-linear with SIC even after Lg transformation and RCS was conducted accordingly. FSI, Framingham steatosis index=-7.981+0.011×age(yrs)-0.146+0.173×early pregnant BMI(kg/m2)+0.007×TG(mg/dL) +0.593×GH (yes=1,no=0)+0.789×GDM(yes=1, no=0)+1.1×[ALT/AST≥1.33(yes=1, no=0)]. Abbreviations: BF%, body fat percentage; SIC, serum iodine concentrations; GMS, gestational metabolic syndromes; PG, post-load glucose level; BMI, body mass index; T1, the first trimester (early pregnancy); GWG, gestational weight gain; TC, total cholesterol; TG, triglycerides; TSH, thyroid stimulating hormone; PCOS, Polycystic Ovary Syndrome; GDM, gestational diabetes mellitus; GH, gestational hypertension; FSI, Framingham steatosis index. FT3/FT4, the ratio of free triiodothyronine to free thyroxine.

Supplemental Table S3 Sensitivity analyses among pregnant women of euthyroid (n=1307), non-gestational hypertension (n=1436), normal pre-pregnancy BMI(n=1337) and normo-lipidemia (n=1152) on the associations of gestational serum iodine concentrations at T1 (LgSIC, μg/L) with metabolic factors using multivariable linear regression models, Huizhou Mother-infant Cohort.

	**Crude model**				**Model 1**				**Model 2 (+ thyroid markers)**		
	B(95% *CI*)	*β*	*P*		*B*(95% *CI*)	*β*	*P*		*B*(95% *CI*)	*β*	*P*
**Women of euthyroid** (n=1307)											
BMI at T1, kg/m^2	-1.032(-3.839,1.775)	-0.020	0.471		-0.999(-3.689, 1.691)	-0.020	0.466		3.743(1.220, 6.267)	0.072	0.004
GWG at T1, kg	-2.371(-4.354, -0.389)	-0.065	0.019		-2.329(-4.313,-0.345)	-0.064	0.021		-0.282(-2.295,1.731)	-0.008	0.784
Lg BF%	0.019(-0.032,0.069)	0.020	0.468		0.023(-0.020,0.065)	0.025	0.292		0.037(-0.007, 0.081)	0.040	0.097
FBG, mmol/L	0.115(-0.145,0.376)	0.024	0.384		0.139(-0.110,0.389)	0.029	0.274		0.162(-0.098, 0.423)	0.034	0.221
1-h PBG, mmol/L	0.572(-0.570,1.713)	0.027	0.326		0.780(-0.311,1.871)	0.037	0.161		1.168(0.038,2.298)	0.056	0.043
2-h PBG, mmol/L	0.856(-0.128,1.840)	0.047	0.088		1.139(0.202,2.077)	0.063	0.017		1.464(0.492, 2.435)	0.081	0.003
HAb1c, %	0.109(-0.010, 0.227)	0.050	0.072		0.118(-0.001,0.236)	0.054	0.052		0.108(-0.015,0.232)	0.049	0.086
TG, mmol/L	0.887(0.467,1.307)	0.114	<0.001		0.977(0.586,1.368)	0.130	<0.001		1.292(0.891.1.692)	0.166	<0.001
TC, mmol/L	0.700(0.210,1.191)	0.077	0.005		0.719(0.230,1.209)	0.079	0.004		0.706(0.196,1.216)	0.078	0.007
TyG-index	0.619(0.356,0.882)	0.127	<0.001		0.682(0.441,0.923)	0.140	<0.001		0.866(0.619, 1.113)	0.177	<0.001
LgTyG-BMI	0.011(-0.044, 0.065)	0.011	0.703		0.032(0.006, 0.059)	0.032	0.016		0.042(0.015, 0.069)	0.041	0.003
LgUA, umol/L	0.108(0.0300.185)	0.076	0.006		0.105(0.031,0.179)	0.074	0.005		0.095(0.015,0.172)	0.067	0.015
FSI	0.506(-0.213,1.225)	0.038	0.168		0.667(0.225,1.109)	0.050	0.003		0.922(0.469,1.375)	0.070	<0.001
**Women without GH** (n=1436)											
BMI at T1	-2.926(-5.382, -0.469)	-0.062	0.020		-2.716(-5.075, -0.357)	-0.057	0.024		2.215(-0.071, 4.501)	0.047	0.058

GWG at T1	-3.807(-5.574,-2.039)	-0.111	<0.001		-3.708(-5.476, -1.939)	-0.108	<0.001		-0.823(-2.651, 1.006)	-0.024	0.378
LgBF%	0.015(-0.032, 0.062)	0.016	0.536		0.031(-0.010, 0.071)	0.034	0.142		0.044(0.000, 0.087)	0.048	0.049
FBG, mmol/L	0.003(-0.228,0.233)	0.001	0.981		0.067(-0.155,0.288)	0.015	0.555		0.115(-0.121,0.351)	0.026	0.338
1-h PBG, mmol/L	0.249(-0.776,1.274)	0.013	0.634		0.535(-0.446, 1.517)	0.027	0.285		0.967(-0.075, 2.009)	0.049	0.069
2-h PBG, mmol/L	0.431(-0.445,1.307)	0.025	0.334		0.717(-0.122,1.556)	0.042	0.094		1.097(0.208,1.987)	0.065	0.016
HbA1c	0.065(-0.039, 0.169)	0.032	0.222		0.078(-0.027, 0.182)	0.039	0.145		0.094(-0.018, 0.205)	0.046	0.100
TG, mmol/L	0.735(0.372,1.099)	0.104	<0.001		0.879(0.540,1.218)	0.125	<0.001		1.200(0.845,1.556)	0.170	<0.001
TC, mmol/L	0.297(-0.138,0.732)	0.035	0.180		0.343(-0.091, 0.777)	0.041	0.122		0.437(-0.025,0.899)	0.052	0.064
TyG-index	0.541(0.312,0.771)	0.121	<0.001		0.641(0.429,0.852)	0.144	<0.001		0.821(0.599,1.043)	0.184	<0.001
Lg TyG-BMI	-0.024(-0.072,0.024)	-0.026	0.331		0.029(0.006, 0.053)	0.032	0.014		0.038(0.013, 0.063)	0.041	0.003
LgUA, umol/L	0.067(-0.001,0.135)	0.051	0.052		0.074(0.008, 0.139)	0.056	0.027		0.065(-0.005, 0.135)	0.049	0.068
FSI	0.228(-0.384,0.840)	0.019	0.465		0.626(0.248,1.004)	0.053	0.001		0.836(0.438, 1.234)	0.071	<0.001
**Women of pre-BMI<23 kg/m^2** (n=1337)											
BMI at T1	-2.950(-4.763, -1.138)	-0.087	0.001		-2.817(-4.608, -1.027)	-0.083	0.002		0.524(-1.256, 2.304)	0.015	0.564
GWG at T1	-3.137(-4.839, -1.434)	-0.098	<0.001		-2.457(-4.108, -0.805)	-0.077	0.004		-0.545(-2.280, 1.191)	-0.017	0.538
Lg BF%	0.016(-0.031, 0.062)	0.018	0.506		0.055(0.015, 0.096)	0.064	0.007		0.053(0.010, 0.096)	0.062	0.016
FBG, mmol/L	-0.081(-0.311, 0.148)	-0.019	0.487		0.019(-0.204, 0.242)	0.004	0.866		0.021(-0.217, 0.259)	0.005	0.172
1-h PBG, mmol/L	0.120(-0.938, 1.177)	0.006	0.824		0.599(-0.418, 1.616)	0.030	0.248		0.853(-0.228, 1.935)	0.043	0.122
2-h PBG, mmol/L	0.302(-0.598, 1.202)	0.018	0.510		0.698(-0.163, 1.559)	0.042	0.112		0.882(-0.034, 1.797)	0.053	0.059
HbA1c	0.038(-0.062, 0.138)	0.021	0.453		0.054(-0.047, 0.154)	0.029	0.297		0.069(-0.039, 0.176)	0.037	0.209

TG, mmol/L	0.602(0.282, 0.922)	0.101	<0.001		0.783(0.477, 1.089)	0.131	<0.001		0.997(0.677, 1.317)	0.167	<0.001
TC, mmol/L	0.403(-0.046, 0.851)	0.048	0.079		0.503(0.054, 0.951)	0.060	0.028		0.544(0.068, 1.020)	0.065	0.025
TyG-index	0.502(0.276,0.727)	0.119	<0.001		0.635(0.422, 0.849)	0.150	<0.001		0.753(0.529, 0.977)	0.178	<0.001
Lg TyG-BMI	-0.022(-0.062,0.017)	-0.030	0.270		0.036(0.017, 0.055)	0.048	<0.001		0.043(0.023, 0.063)	0.058	<0.001
LgUA, umol/L	0.058(-0.011, 0.127)	0.045	0.102		0.074(0.006, 0.143)	0.058	0.033		0.054(-0.019, 0.127)	0.042	0.144
FSI	0.098(-0.424, 0.621)	0.010	0.712		0.769(0.412, 1.126)	0.079	<0.001		0.788(0.411, 1.164)	0.081	<0.001
**Women of normo-lipidemia** (n=1152)											
BMI at T1	-4.068(-6.604, -1.532)	-0.092	0.002		-3.835(-6.314, -1.355)	-0.087	0.002		0.207(-2.251, 2.664)	0.005	0.869
GWG at T1	-4.134(-6.001, -2.266)	-0.127	<0.001		-4.400(-6.275,-2.525)	-0.135	<0.001		-1.814(-3.788,0.160)	-0.056	0.072
Lg BF%	-0.014(-0.066,0.039)	-0.015	0.607		0.011(-0.035,0.057)	0.012	0.632		0.017(-0.032, 0.066)	0.019	0.493
FBG, mmol/L	-0.036(-0.278,0.205)	-0.009	0.767		0.049 (-0.186,0.284)	0.012	0.683		0.058(-0.196,0.312)	0.014	0.654
1-h PBG, mmol/L	0.292(-0.823,1.406)	0.015	0.608		0.751(-0.335,1.837)	0.039	0.175		1.067(-0.101,2.235)	0.055	0.073
2-h PBG, mmol/L	0.445(-0.488,1.377)	0.028	0.350		0.849(-0.058,1.755)	0.053	0.067		1.052(0.077,2.027)	0.065	0.035
HbA1c	0.058(-0.055,0.170)	0.030	0.315		0.077(-0.036, 0.190)	0.040	0.182		0.086(-0.036, 0.208)	0.044	0.167
TG (mmol/L)	0.537(0.336,0.737)	0.153	<0.001		0.624(0.430,0.818)	0.178	<0.001		0.696(0.488,0.903)	0.198	<0.001
TC (mmol/L)	0.115(-0.280,0.511)	0.017	0.568		0.149(-0.247,0.545)	0.022	0.460		0.250(-0.176,0.675)	0.037	0.250
TyG-index	0.452(0.260,0.644)	0.135	<0.001		0.540(0.355,0.725)	0.161	<0.001		0.584(0.385,0.783)	0.174	<0.001
LgTyG-BMI	-4.068(-6.604, -1.532)	-0.092	0.002		-3.835(-6.314, -1.355)	-0.087	0.002		0.207(-2.251, 2.664)	0.005	0.869
LgUric acid (umol/L)	0.073(-0.004,0.150)	0.055	0.062		0.083(0.008,0.157)	0.062	0.030		0.067(-0.014, 0.147)	0.050	0.103
FSI	0.078(-0.495,0.652)	0.008	0.789		0.650(0.321, 0.978)	0.066	<0.001		0.669(0.316, 1.022)	0.068	<0.001

Mothers of diagnosed pre-pregnancy hepatic or thyroid disorders or current hepatic diseases were excluded. Serum iodine concentrations (SIC), body fat%(BF), TyG-BMI, and uric acid (UA) were Log10 transformed due to skewed distribution. Data were analyzed by multivariable linear regression with covariates being adjusted by enter method. The adjusted covariates in model 1 included, maternal age(y), pre-pregnancy BMI(kg/m^2), education (primary school and below, middle school, college, university and above), parity (0, 1,2, ≥3), smoking (yes/no), alcohol drinking (yes/no), medical history of GDM (yes/no), pre-pregnancy PCOS or thalassemia (yes/no), first-degree family history of diabetes(yes/no), hormone usage (yes/no), folate supplementation(yes/no), total sitting time(hrs/d); In model 2, further adjustment was made for thyroid hormones including LgTSH and LgFT3/FT4. For BMI at T1, GWG at T1 and TyG-BMI, pre-pregnancy BMI was not adjusted in the multivariable linear regression model to avoid possible collinearity.
TyG-index=In[TG (mg/dL)×FPG (mg/dL) /2]; TyG-BMI= TyG-index×BMI at T1;   FSI=-7.981+0.011×age(yrs)-0.146+0.173×early pregnancy BMI(kg/m^2)+0.007×TG(mg/dL)+0.593×GH(yes=1,no=0)+0.789×GDM(yes=1, no=0)+1.1×[ALT/AST≥1.33(yes=1, no=0)];
Abbreviations: B, unstandardized coefficient; β, standardized coefficient; BF%, body fat percentage; SIC, serum iodine concentrations; GMS, gestational metabolic syndromes; BMI, body mass index; T1, the first trimester (early pregnancy); GWG, gestational weight gain; TC, total cholesterol; TG, triglycerides; TSH, thyroid stimulating hormone; PCOS, Polycystic Ovary Syndrome; GDM, gestational diabetes mellitus; GH, gestational hypertension; FSI, Framingham steatosis index. FT3/FT4, the ratio of free triiodothyronine to free thyroxine.

Supplemental Table S4 Subgroup analyses by GDM status on the associations of maternal SIC at T1 with metabolic factors by multivariable linear regression models, Huizhou mother-infant cohort.

	**Crude model**				**Model 1**				**Model 2 (+ thyroid markers)**		
	B(95% *CI*)	*β*	*P*		*B*(95% *CI*)	*β*	*P*		*B*(95% *CI*)	*β*	*P*
**Non-GDM mothers** (n=1195)											
**GWG at T1**	-3.655(-5.392,-1.918)	-0.119	<0.001		-3.003(-4.694,-1.313)	-0.098	0.001		-0.948(-2.724,0.828)	-0.031	0.295
**FBG, mmol/L**	-0.183(-0.373,0.007)	-0.055	0.059		-0.131(-0.320,0.058)	-0.039	0.175		-0.135(-0.337,0.067)	-0.040	0.191
**1-h PBG, mmol/L**	-0.115(-0.981,0.750)	-0.008	0.794		0.239(-0.631,1.091)	0.016	0.582		0.449(-0.460,1.358)	0.029	0.333
**2-h PBG, mmol/L**	0.139(-0.534,0.813)	0.012	0.684		0.369(-0.296,1.033)	0.031	0.276		0.500(-0.209,1.209)	0.042	0.167
**TG (mmol/L)**	0.463(0.106,0.820)	0.074	0.011		0.688(0.350,1.026)	0.109	<0.001		0.907(0.550,1.265)	0.144	<0.001
**TC (mmol/L)**	0.368(-0.099,0.835)	0.045	0.123		0.491(0.026,0.956)	0.060	0.039		0.590(0.095,1.085)	0.072	0.020
**TyG-index**	0.412(0.185,0.640)	0.102	<0.001		0.562(0.349,0.776)	0.140	<0.001		0.680(0.455,0.906)	0.169	<0.001
**TyG-BMI**	-18.756(-40.261,2.749)	-0.049	0.087		12.851(4.481,21.220)	0.034	0.003		14.035(5.131,22.939)	0.037	0.002
**LgUric acid**	0.058(-0.015,0.132)	0.045	0.120		0.083(0.012,0.154)	0.064	0.023		0.062(-0.014,0.139)	0.048	0.108
**FSI**	-0.230(-0.799,0.339)	-0.023	0.427		0.555(0.253,0.857)	0.055	<0.001		0.558(0.238,0.878)	0.056	0.001
**GDM mothers** (n=245)											
**GWG at T1**	-5.340(-11.153,0.472)	-0.115	0.072		-4.673(-10.420,1.074)	-0.101	0.110		-3.300(-9.420,2.819)	-0.071	0.289
**FBG, mmol/L**	0.907(-0.020,1.833)	0.123	0.055		1.011(0.138,1.885)	0.137	0.023		1.054(0.118,1.991)	0.143	0.028
**1-h PBG, mmol/L**	1.426(-1.243,4.095)	0.067	0.294		1.569(-1.101,4.240)	0.074	0.248		2.039(-0.819,4.898)	0.096	0.161
**2-h PBG, mmol/L**	1.569(-0.890,4.028)	0.080	0.210		1.964(-0.503,4.430)	0.101	0.118		2.822(0.198,5.446)	0.145	0.035
**TG (mmol/L)**	1.373(0.135,2.610)	0.139	0.030		1.715(0.540,2.889)	0.173	0.004		2.310(1.086,3.534)	0.234	<0.001
**TC (mmol/L)**	-0.099(-1.281,1.082)	-0.011	0.868		0.020(-1.176,1.216)	0.002	0.974		-0.314(-1.586,0.958)	-0.034	0.627

**TyG-index**	0.919(0.175,1.662)	0.154	0.016		1.152(0.483,1.821)	0.193	0.001		1.470(0.772,2.169)	0.247	<0.001
**TyG-BMI**	7.651(-63.809,78.931)	0.013	0.835		30.450(11.390,49.510)	0.054	0.002		40.338(20.334,60.343)	0.071	<0.001
**LgUric acid**	0.067(-0.106,0.241)	0.049	0.444		0.070(-0.100,0.240)	0.051	0.418		0.048(-0.134,0.229)	0.035	0.605
**FSI**	1.038(-0.710,2.785)	0.075	0.243		1.609(0.593,2.625)	0.116	0.002		1.856(0.787,2.926)	0.134	0.001

Mothers of diagnosed pre-pregnancy hepatic or thyroid disorders or current hepatic diseases were excluded for analysis. Serum iodine concentrations (SIC) were Log10 transformed due to skewed distribution. Data were analyzed by multivariable linear regression with covariates being adjusted by enter method. The adjusted covariates in model 1 included, maternal age(y), education (primary school and below, middle school, college, university and above), parity (0, 1,2, ≥3), smoking (yes/no), alcohol drinking (yes/no), medical history of GDM (yes/no), pre-pregnancy PCOS or thalassemia (yes/no), first-degree family history of diabetes(yes/no); model 2 further adjustment for thyroid hormones including LgTSH and LgFT3/FT4. For BMI at T1, GWG at T1 and TyG-BMI, pre-pregnancy BMI was not adjusted in the multivariable linear regression model to avoid possible collinearity.

TyG-index=In[TG (mg/dL)×FPG (mg/dL) /2]; TyG-BMI= TyG-index×BMI at T1; FSI=-7.981+0.011×age(yrs)-0.146+0.173×BMI at T1 (kg/m^2)+0.007×TG(mg/dL)+0.593×GH(yes=1,no=0)+0.789×GDM(yes=1, no=0)+1.1×[ALT/AST≥1.33(yes=1, no=0)];

Abbreviations: B, unstandardized coefficient; β, standardized coefficient; BF%, body fat percentage; SIC, serum iodine concentrations; GMS, gestational metabolic syndromes; BMI, body mass index; T1, the first trimester (early pregnancy); GWG, gestational weight gain; TC, total cholesterol; TG, triglycerides; TSH, thyroid stimulating hormone; PCOS, Polycystic Ovary Syndrome; GDM, gestational diabetes mellitus; GH, gestational hypertension; FSI, Framingham steatosis index. FT3/FT4, the ratio of free triiodothyronine to free thyroxine.

Supplemental Table S5 Subgroup analyses by TyG-index of < or ≥8.73 on the associations of maternal SIC at T1 with metabolic factors by multivariable linear regression models, Huizhou mother-infant cohort.

	**Crude model**				**Model 1**				**Model 2 (+ thyroid markers)**		
	B(95% *CI*)	*β*	*P*		*B*(95% *CI*)	*β*	*P*		*B*(95% *CI*)	*β*	*P*
**TyG-index<8.73** (n=1092)											
**BMI at T1**	-3.963(-6.443, -1.482)	-0.095	0.002		-3.945(-6.383, -1.507)	-0.094	0.002		-0.007(-2.425, 2.411)	-0.005	0.996
**GWG at T1**	-2.917 (-4.889, -0.946)	-0.088	0.004		-3.140(-5.128, -1.153)	-0.094	0.002		-0.756(-2.849, 1.336)	-0.023	0.478
**FBG, mmol/L**	-0.224(-0.461, 0.013)	-0.056	0.064		-0.145(-0.376, 0.086)	-0.036	0.219		-0.113(-0.360, 0.135)	-0.028	0.372
**1-h PBG, mmol/L**	-0.214(-1.389, 0.962)	-0.011	0.721		0.284(-0.855, 1.422)	0.014	0.625		0.711(-0.503, 1.924)	0.036	0.251
**2-h PBG, mmol/L**	-0.003(-0.984, 0.978)	0.000	0.995		0.437(-0.508, 1.381)	0.026	0.364		0.768(-0.238, 1.774)	0.047	0.135
**TG (mmol/L)**	0.495(0.288, 0.702)	0.141	<0.001		0.586(0.383, 0.789)	0.167	<0.001		0.692(0.476, 0.907)	0.197	<0.001
**TC (mmol/L)**	0.106(-0.383, 0.595)	0.013	0.671		0.151(-0.340, 0.642)	0.018	0.546		0.185(-0.338, 0.708)	0.022	0.488
**TyG-index**	0.446(0.242,0.650)	0.129	<0.001		0.549(0.351, 0.746)	0.158	<0.001		0.651(0.441, 0.861)	0.188	<0.001
**Lg TyG-BMI**	-0.055(-0.107,-0.003)	-0.062	0.040		0.004(-0.017, 0.024)	0.004	0.734		0.033(0.012,0.055)	0.038	0.002
**LgUric acid**	0.043(-0.035, 0.121)	0.033	0.281		0.052(-0.024, 0.129)	0.040	0.178		0.036(-0.046, 0.118)	0.027	0.387
**FSI**	-0.187(-0.770, 0.396)	-0.019	0.530		0.510(0.206, 0.814)	0.052	0.001		0.640(0.317, 0.964)	0.065	<0.001
**TyG-index≥8.73** (n=204)											
**BMI at T1**	-6.243(-15.080, 2.594)	-0.107	0.165		-6.778(-15.485, 1.929)	-0.116	0.126		1.967(-6.846, 10.779)	0.034	0.660
**GWG at T1**	-11.334(-17.045,-	-0.288	<0.001		-9.703(-15.145, -4.261)	-0.247	0.001		-4.424(-10.000, 1.152)	-0.113	0.119
**FBG, mmol/L**	1.322(0.254, 2.390)	0.185	0.016		1.469(0.382, 2.556)	0.205	0.008		1.337(0.146, 2.528)	0.187	0.028
**1-h PBG, mmol/L**	1.938(-1.925, 5.801)	0.076	0.323		2.306(-1.489, 6.100)	0.090	0.232		2.560(-1.599, 6.720)	0.100	0.226
**2-h PBG, mmol/L**	1.272(-2.283, 4.827)	0.054	0.481		1.676(-1.831,5.183)	0.071	0.347		1.920(-1.916, 5.756)	0.082	0.324
**TG (mmol/L)**	-0.555(-2.321, 1.211)	-0.048	0.536		-0.443(-2.312, 1.425)	-0.038	0.640		0.421(-1.591, 2.433)	0.036	0.680
**TC (mmol/L)**	0.523(-0.981, 2.028)	0.053	0.493		0.591(-0.991, 2.173)	0.060	0.462		0.637(-1.092, 2.367)	0.064	0.468
**TyG-index**	0.062(-0.505, 0.629)	0.017	0.829		0.115(-0.479, 0.708)	0.031	0.704		0.481(-0.151, 1.113)	0.129	0.135
**Lg TyG-BMI**	-0.119(-0.278, 0.040)	-0.113	0.141		-0.070(-0.122, -0.018)	-0.067	0.009		-0.009(-0.060, 0.042)	-0.008	0.733

**LgUric acid**	0.080(-0.127, 0.286)	0.058	0.448		0.067(-0.129, 0.262)	0.049	0.500		0.039(-0.175, 0.253)	0.029	0.361
**FSI**	-1.234(-3.569, 1.102)	-0.080	0.299		-0.683(-2.237, 0.871)	-0.044	0.387		-0.144(-1.798, 1.511)	-0.009	0.864

Mothers of diagnosed pre-pregnancy hepatic or thyroid disorders or current hepatic diseases were excluded for analysis. Serum iodine concentrations (SIC) were Log10 transformed due to skewed distribution. Data were analyzed by multivariable linear regression with covariates being adjusted by enter method. The adjusted covariates in model 1 included, maternal age(y), education (primary school and below, middle school, college, university and above), parity (0, 1,2, ≥3), smoking (yes/no), alcohol drinking (yes/no), medical history of GDM (yes/no), pre-pregnancy PCOS or thalassemia (yes/no), first-degree family history of diabetes(yes/no); model 2 further adjustment for thyroid hormones including LgTSH and LgFT3/FT4. For BMI at T1, GWG at T1 and TyG-BMI, pre-pregnancy BMI was not adjusted in the multivariable linear regression model to avoid possible collinearity.

TyG-index=In[TG (mg/dL)×FPG (mg/dlL) /2]; TyG-BMI= TyG-index×BMI at T1; FSI=-7.981+0.011×age(yrs)-0.146+0.173×BMI at T1(kg/m^2)+0.007×TG(mg/dL)+0.593×GH(yes=1,no=0)+0.789×GDM(yes=1, no=0)+1.1×[ALT/AST≥1.33(yes=1, no=0)];

Abbreviations: B, unstandardized coefficient; β, standardized coefficient; BF%, body fat percentage; SIC, serum iodine concentrations; GMS, gestational metabolic syndromes; BMI, body mass index; T1, the first trimester (early pregnancy); GWG, gestational weight gain; TC, total cholesterol; TG, triglycerides; TSH, thyroid stimulating hormone; PCOS, Polycystic Ovary Syndrome; GDM, gestational diabetes mellitus; GH, gestational hypertension; FSI, Framingham steatosis index. FT3/FT4, the ratio of free triiodothyronine to free thyroxine.

Supplemental Table S6 Subgroup analyses by parity on the associations of maternal SIC at T1 with metabolic factors by multivariable linear regression models, Huizhou mother-infant cohort.

	**Crude model**				**Model 1**				**Model 2 (+ thyroid markers)**		
	B(95% *CI*)	*β*	*P*		*B*(95% *CI*)	*β*	*P*		*B*(95% *CI*)	*β*	*P*
**Nulliparity**（n=800）											
GWG at T1	-3.274(-5.640,-0.907)	-0.096	0.007		-2.357(-4.607,-0.106)	-0.069	0.040		-0.488(-2.859,1.884)	-0.014	0.687
FBG, mmol/L	-0.049(-0.371,0.272)	-0.011	0.764		0.133(-0.180,0.447)	0.029	0.403		0.166(-0.168,0.500)	0.036	0.329
1-h PBG, mmol/L	1.354(-0.047,2.755)	0.067	0.058		2.145(0.772,3.517)	0.106	0.002		2.407(0.949,3.866)	0.119	0.001
2-h PBG, mmol/L	1.200(-0.023,2.424)	0.068	0.054		1.785(0.577,2.993)	0.101	0.004		2.115(0.833,3.396)	0.120	0.001
TG (mmol/L)	0.585(0.105,1.064)	0.084	0.017		0.858(0.405,1.311)	0.124	<0.001		1.144(0.667,1.621)	0.165	<0.001
TC (mmol/L)	0.164(-0.408,0.747)	0.020	0.580		0.325(-0.258,0.908)	0.039	0.274		0.340(-0.281,0.960)	0.041	0.283
TyG-index	0.464(0.161,0.767)	0.106	0.003		0.654(0.364,0.943)	0.149	<0.001		0.825(0.521,1.128)	0.188	<0.001
TyG-BMI	-20.180(-	-0.051	0.153		14.459(1.174,27.744)	0.036	0.033		18.664(4.571,32.756)	0.047	0.010
LgUA (umol/L)	0.053(-0.037,0.143)	0.041	0.248		0.075(-0.011,0.160)	0.057	0.086		0.035(-0.055,0.126)	0.027	0.442
FSI	-0.401(-1.216,0.415)	-0.034	0.335		0.559(0.022,1.095)	0.048	0.041		0.628(0.060,1.195)	0.053	0.030
**Multiparity**（n=666）											
GWG at T1	-4.611(-7.237,-1.985)	-0.133	0.001		-4.182(-6.744,-1.620)	-0.120	0.001		-1.861(-4.558,0.837)	-0.054	0.176
FBG, mmol/L	0.099(-0.222,0.420)	0.024	0.543		0.127(-0.180,0.433)	0.030	0.417		0.110(-0.218,0.438)	0.026	0.512
1-h PBG, mmol/L	-1.035(-2.527,0.457)	-0.053	0.174		-0.806(-2.184,0.572)	-0.041	0.251		-0.413(-1.884,1.058)	-0.021	0.582
2-h PBG, mmol/L	-0.404(-1.638,0.830)	-0.025	0.521		-0.240(-1.376,0.897)	-0.015	0.679		0.032(-1.181,1.244)	0.002	0.959
TG (mmol/L)	0.800(0.248,1.352)	0.110	0.005		0.877(0.362,1.391)	0.120	0.001		1.150(0.608,1.692	0.158	<0.001
TC (mmol/L)	0.472(-0.170,1.114)	0.056	0.149		0.416(-0.220,1.052)	0.049	0.199		0.484(-0.193,1.160)	0.057	0.161
TyG-index	0.601(0.263,0.940)	0.134	0.001		0.651(0.343,0.960)	0.145	<0.001		0.778(0.452,1.104)	0.174	<0.001
TyG-BMI	0.145(-33.366,33.656)	0.000	0.993		12.623(-2.963,28.209)	0.029	0.112		12.835(-3.755,29.425)	0.029	0.129
LgUric acid	0.077(-0.026,0.180)	0.057	0.142		0.084(-0.016,0.184)	0.062	0.099		0.086(-0.021,0.193)	0.063	0.116
FSI	0.727(-0.233,1.688)	0.058	0.137		1.054(0.472,1.636)	0.084	<0.001		1.058(0.442,1.675)	0.084	0.001

Mothers of diagnosed pre-pregnancy hepatic or thyroid disorders or current hepatic diseases were excluded for analysis. Serum iodine concentrations (SIC) were Log10 transformed due to skewed distribution. Data were analyzed by multivariable linear regression with covariates being adjusted by enter method. The adjusted covariates in model 1 included, maternal age(y), education (primary school and below, middle school, college, university and above), parity (0, 1,2, ≥3), smoking (yes/no), alcohol drinking (yes/no), medical history of GDM (yes/no), pre-pregnancy PCOS or thalassemia (yes/no), first-degree family history of diabetes(yes/no); model 2 further adjustment for thyroid hormones including LgTSH and LgFT3/FT4. For BMI at T1, GWG at T1 and TyG-BMI, pre-pregnancy BMI was not adjusted in the multivariable linear regression model to avoid possible collinearity.

TyG-index=In[TG (mg/dL)×FPG (mg/dL) /2]; TyG-BMI= TyG-index×BMI at T1; FSI=-7.981+0.011×age(yrs)-0.146+0.173×BMI at T1(kg/m^2)+0.007×TG(mg/dL)+0.593×GH(yes=1,no=0)+0.789×GDM(yes=1, no=0)+1.1×[ALT/AST≥1.33(yes=1, no=0)];

Abbreviations: B, unstandardized coefficient; β, standardized coefficient; BF%, body fat percentage; SIC, serum iodine concentrations; GMS, gestational metabolic syndromes; BMI, body mass index; T1, the first trimester (early pregnancy); GWG, gestational weight gain; TC, total cholesterol; TG, triglycerides; TSH, thyroid stimulating hormone; PCOS, Polycystic Ovary Syndrome; GDM, gestational diabetes mellitus; GH, gestational hypertension; FSI, Framingham steatosis index. FT3/FT4, the ratio of free triiodothyronine to free thyroxine.
